# Continuous Versus Intermittent Vital Signs Monitoring Using a Wearable, Wireless Patch in Patients Admitted to Surgical Wards: Pilot Cluster Randomized Controlled Trial

**DOI:** 10.2196/10802

**Published:** 2018-12-11

**Authors:** Candice Downey, Rebecca Randell, Julia Brown, David G Jayne

**Affiliations:** 1 Leeds Institute of Biomedical & Clinical Sciences University of Leeds Leeds United Kingdom; 2 School of Healthcare University of Leeds Leeds United Kingdom; 3 Leeds Institute of Clinical Trials Research University of Leeds Leeds United Kingdom

**Keywords:** general surgery, monitoring, physiological, randomized controlled trial, vital signs

## Abstract

**Background:**

Vital signs monitoring is a universal tool for the detection of postoperative complications; however, unwell patients can be missed between traditional observation rounds. New remote monitoring technologies promise to convey the benefits of continuous monitoring to patients in general wards.

**Objective:**

The aim of this pilot study was to evaluate whether continuous remote vital signs monitoring is a practical and acceptable way of monitoring surgical patients and to optimize the delivery of a definitive trial.

**Methods:**

We performed a prospective, cluster-randomized, parallel-group, unblinded, controlled pilot study. Patients admitted to 2 surgical wards at a large tertiary hospital received either continuous and intermittent vital signs monitoring or intermittent monitoring alone using an early warning score system. Continuous monitoring was provided by a wireless patch, worn on the patient’s chest, with data transmitted wirelessly every 2 minutes to a central monitoring station or a mobile device carried by the patient’s nurse. The primary outcome measure was time to administration of antibiotics in sepsis. The secondary outcome measures included the length of hospital stay, 30-day readmission rate, mortality, and patient acceptability.

**Results:**

Overall, 226 patients were randomized between January and June 2017. Of 226 patients, 140 were randomized to continuous remote monitoring and 86 to intermittent monitoring alone. On average, patients receiving continuous monitoring were administered antibiotics faster after evidence of sepsis (626 minutes, n=22, 95% CI 431.7-820.3 minutes vs 1012.8 minutes, n=12, 95% CI 425.0-1600.6 minutes), had a shorter average length of hospital stay (13.3 days, 95% CI 11.3-15.3 days vs 14.6 days, 95% CI 11.5-17.7 days), and were less likely to require readmission within 30 days of discharge (11.4%, 95% CI 6.16-16.7 vs 20.9%, 95% CI 12.3-29.5). Wide CIs suggest these differences are not statistically significant. Patients found the monitoring device to be acceptable in terms of comfort and perceived an enhanced sense of safety, despite 24% discontinuing the intervention early.

**Conclusions:**

Remote continuous vital signs monitoring on surgical wards is practical and acceptable to patients. Large, well-controlled studies in high-risk populations are required to determine whether the observed trends translate into a significant benefit for continuous over intermittent monitoring.

**Trial Registration:**

International Standard Randomised Controlled Trial Number ISRCTN60999823; http://www.isrctn.com /ISRCTN60999823 (Archived by WebCite at http://www.webcitation.org/73ikP6OQz)

## Introduction

Perioperative complications are unfortunately common following surgical procedures. Postoperative mortality is the third leading cause of death in the United States [1]. The International Surgical Outcomes Study found that 17% of patients undergoing inpatient surgery developed at least one complication [2]. This figure rose to 27% in patients undergoing major surgery. In addition, 2.8% of patients who developed a postoperative complication died before discharge from hospital. Monitoring patients beyond the operating room is important to allow early detection of clinical deterioration and timely intervention [3].

The early warning score system is predicated on the idea that derangements in simple physiological observations can identify hospital inpatients at high risk of deterioration [4]. Prodromal warning signs, such as increased respiratory rate or decreased blood pressure, precede critical illness [5], allowing early recognition and management of patients to reverse the abnormal physiological decline or prompt admission to a critical care area.

A critical limitation of early warning score systems is their intermittent nature [6]. Clinical deterioration on general wards may remain undetected for hours before clinicians are alerted [3]. One solution may be continuous vital signs monitoring, which until now has been limited to use on critical care wards owing to prohibitive cost and implications for patient mobility and recovery.

The development of wireless and wearable sensors allows continuous monitoring of ambulatory patients. A number of such tools have already received the Food and Drug Administration clearance, but clinical studies are required to demonstrate their clinical utility in the postsurgical setting [3,7].

A recent systematic review identified 9 studies assessing the effect of continuous vital signs monitoring on general wards [8]. The authors found no evidence of a marked reduction in intensive care unit transfers or other adverse events with continuous monitoring but recognized heterogeneous methods, study populations, and outcome measures. Efficient, well-designed pilot studies are vital to ensure the robust design and implementation of large-scale clinical trials [9].

This study aims to evaluate whether continuous remote vital signs monitoring is a practical way of monitoring surgical patients outside of the critical care setting and whether its use is acceptable to patients. The pilot data will be used to inform a further definitive trial to optimize recruitment, treatment compliance, and follow-up protocols.

## Methods

### Study Design

The study was designed as a pilot cluster-randomized, prospective, parallel-group, controlled single-center pilot study, comparing remote continuous vital signs monitoring and intermittent monitoring with intermittent monitoring alone.

Ethical approval was granted on November 30, 2016, by the Yorkshire & The Humber—Bradford Leeds Research Ethics Committee (ref: 16/YH/0426). The study was prospectively registered on the International Standard Randomised Controlled Trial Number registry (ISRCTN60999823). No changes were made to the registered protocol. The trial was performed in accordance with the Good Clinical Practice guidelines and the Declaration of Helsinki and is presented according to the Consolidated Standards of Reporting Trials (CONSORT) statement principles [10] and the CONSORT-EHEALTH checklist (Multimedia Appendix 1) [11].

The study population comprised patients admitted to 2 elective general surgery wards (male and female) at a single tertiary center in Leeds, United Kingdom. Patients aged ≥18 years who were able to provide informed consent to participate were included. Patients with a known allergy to the electrode adhesive and those with a cardiac pacemaker *in situ* were excluded. Patients were approached face-to-face by a research nurse or clinical fellow as soon as possible after their admission onto the wards. After consideration of a patient information sheet (see Multimedia Appendix 2), participants gave informed consent to enter the study.

### Randomization

Consenting participants were allocated to one of the two monitoring arms for the length of their admission, according to the ward bay they were first arbitrarily admitted to. Each ward has 4 bays, containing 6 beds each.

Of the 4 bays on each ward, 3 were randomly allocated to one of the monitoring arms; 2 bays were allocated to receive the patch and one to receive usual intermittent monitoring. Each bay was independently block randomized to an intervention arm by the primary investigator (CD) using Web-based software: Sealed Envelope [12].

The 2 remaining bays (one on each ward) could not be randomized because they did not have the required hardware installed. Patients in these bays were therefore allocated to receive usual intermittent monitoring alone.

The allocation of patients to each bay was performed by hospital bed managers, who were independent of the trial and unaware of the bay allocations. Owing to the nature of the intervention, neither patients nor their nurses were blinded to the allocated monitoring arm.

### Control

All patients in the study received usual intermittent vital signs monitoring. In our institution, this is the National Early Warning Score, which involves intermittent manual charting of vital signs and the calculation of a combined score, indicating the patient’s status. The control arm received intermittent monitoring alone. For postoperative patients, this typically consisted of an hourly recording of blood pressure, pulse, temperature, respiratory rate, and oxygen saturation until the patient's condition was stable when the frequency of observations was decreased to 2 hourly and, then, 4 hourly. For patients not undergoing an operation, the frequency of monitoring was tailored to their condition.

### Intervention

**Figure 1:**
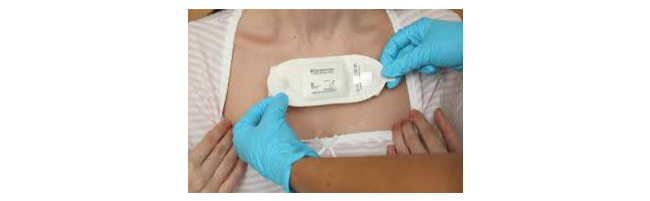
The SensiumVitals patch. Photograph used with permission from Sensium (Abingdon, United Kingdom).

Patients admitted to an intervention bay received usual intermittent monitoring, in addition to continuous vital signs monitoring through the SensiumVitals (Sensium, Abingdon, United Kingdom) system. This system consists of a Conformité Européene–marked wireless patch (Figure 1) worn on the chest of a patient, which continuously monitors heart rate, respiratory rate, and temperature. The data are transmitted wirelessly every 2 minutes to a central monitoring station or a mobile device carried by the patient’s nurse. The nurse is alerted when there is any deviation from preset physiological norms. The alert prompts an acknowledgment of the notification, after which nurses are free to act according to their clinical discretion. Reminders were sent every 14 minutes until acknowledgment, and levels of engagement were monitored through daily ward visits. All other clinical care remained as normal in the intervention group.

The monitoring system was set up in the wards over a period of 6 weeks, during which a number of stakeholders were engaged with the project. Early on, permission from the Estates and Information Technology departments was obtained. The ceiling-mounted bridges were installed by the hospital Estates department using existing electrical wiring circuits to ensure compliance with local policies. The monitoring software was integrated with the hospital admissions data system so that patients could easily be added to the remote monitoring system. All data were stored and retained on the hospital network, alleviating initial concerns about data security by inheriting all hospital security procedures and data backup policies.

General surgeons were informed of the project at local audit meetings so that they would understand potential escalations of care prompted by the device, although they were not expected to apply the patches or carry the mobile devices. Nursing staff were trained face-to-face to use the system over a period of 1 week, after which ad-hoc refresher training was available on request.

### Primary Outcome Measure

The primary outcome measure was time to antibiotics after the first evidence of sepsis, defined according to a revised consensus conference definition in 2001 by the presence of a likely source of infection and ≥2 of the following criteria [13]: (1) temperature >38.3°C or <36.0°C; (2) tachycardia >90 beats/minute; (3) tachypnea >20 breaths/minute; (4) partial pressure of carbon dioxide <4.3 kPa; (5) hyperglycemia (blood glucose >6.6 mmol/L) in the absence of diabetes mellitus; (6) acutely altered mental status; and (7) white blood cells count>12×109/L or <4×109/L.

The decision to prescribe antibiotics was usually made by a junior doctor on the ward, based on local protocols and clinical discretion. The time to antibiotics was determined by review of the observations chart, SensiumVitals data, electronic medications record, and medical notes of patients during their hospital admission.

### Secondary Outcome Measures

The secondary outcome measures were in-hospital mortality, length of hospital stay, number of admissions to Level II or III care, and readmission to hospital within 30 days of discharge.

### Patient Acceptability and Compliance

Patient compliance was determined by the number of patients not wearing a patch for at least 5 days. To assess the acceptability, patients in the continuous monitoring group were asked to complete a short 2-question questionnaire at the bedside on the day of discharge from hospital. Patients were asked to rank the comfort and sense of safety they perceived from wearing the patch on a scale from “Strongly Agree” to “Strongly Disagree.”

### Data Collection

The data collection was performed daily by a research nurse and a clinical fellow. This allowed any harms or benefits to be collected in real time. The data were taken from the clinical records made by patients’ usual care teams, including a succession of junior medical staff on rotation, who were unaware of the study. The objective methods of collecting the outcome data minimized the risk of bias. In addition, the predefined criteria for the outcome measures provided minimal scope for interpretation of their presence or absence by the data collection team.

### Statistical Analysis

A formal sample size calculation was not possible given the lack of data surrounding the primary outcome measure; thus, assumptions were used to calculate an appropriate sample size. A sample size of 325-625 was suggested as an appropriate target based on the assumed eligibility rate (90%), consent rate (30%-50%), and patient turnover (4 patients per bed per calendar month).

The analysis was on an intention-to-treat basis at the individual patient level. Each of the outcome measures was summarized by the intervention or control group using descriptive statistics. As there was no formal sample size calculation, no statistical comparison between trial arms was made.

### Exploratory Analysis

The primary analysis included only the 6 randomized bays. The 2 nonrandomized bays were included in a separate exploratory analysis.

### Progression Criteria

Although no formal progression criteria were defined in the protocol, considerations for the progression to a definitive trial included:

- Recruitment rate (at least 325 patients within the 9-month recruitment period)
- Protocol adherence (proportion of patients wearing the patch for at least 5 days)
- Suitability of primary outcome measure to inform the sample size of a definitive trial.

## Results

### Principal Results

**Figure 2:**
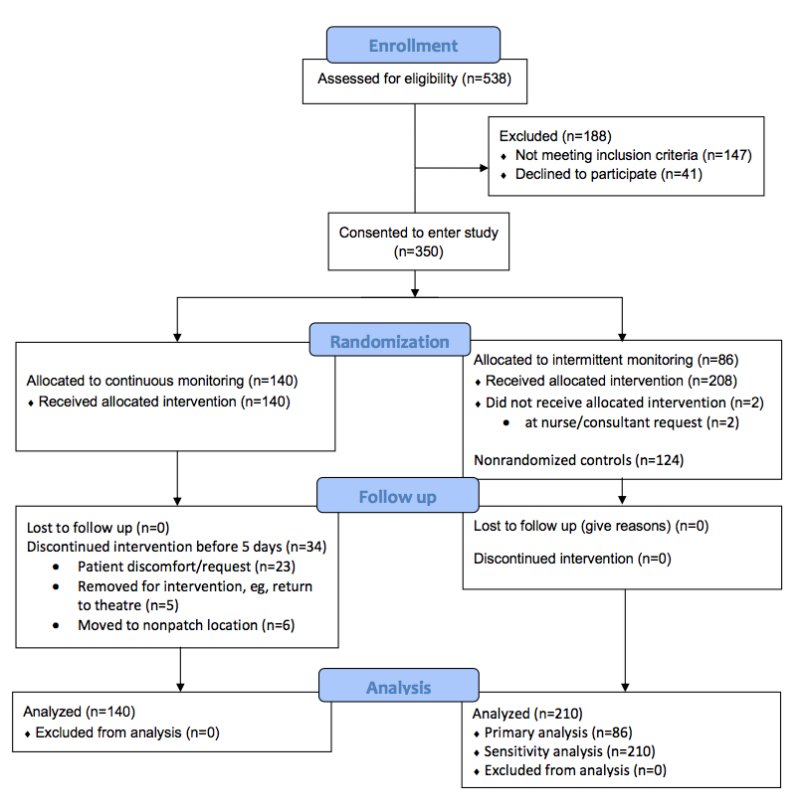
The Consolidated Standards of Reporting Trials flow diagram for the trial.

In this study, 226 patients were randomized between January and June 2017. Figure 2 presents the patient flowchart, and Table 1 presents patients’ characteristics.

While 140 patients were allocated to receive continuous monitoring alongside standard care, 86 patients were randomized to the control group. A further 124 patients from nonrandomized bays were included in the exploratory analysis.

Two patients in the control arm (both from randomized bays) were given the continuous monitoring intervention at the request of the direct care team.

Overall, 73% (257/350) of patients underwent a surgical intervention during their admission; these were mostly colorectal resections (n=132), stoma formations (n=23), stoma reversals (n=12), hernia repairs (n=20), and other colorectal laparotomies, including fistula exploration (n=23). Less common procedures were hepatobiliary (n=14), urological (n=9), appendicectomy (n=7), and abdominal wall repair (n=5). Of note, 8 procedures were classified as Other.

A similar number of complications and sepsis events occurred across both arms of the study (see Multimedia Appendix 3), indicating that both groups had similar baseline risk factors.

One patient died of alcoholic liver disease during their participation in the study. This patient was allocated to receive continuous monitoring.

### Primary Outcome Measure

**Figure 3:**
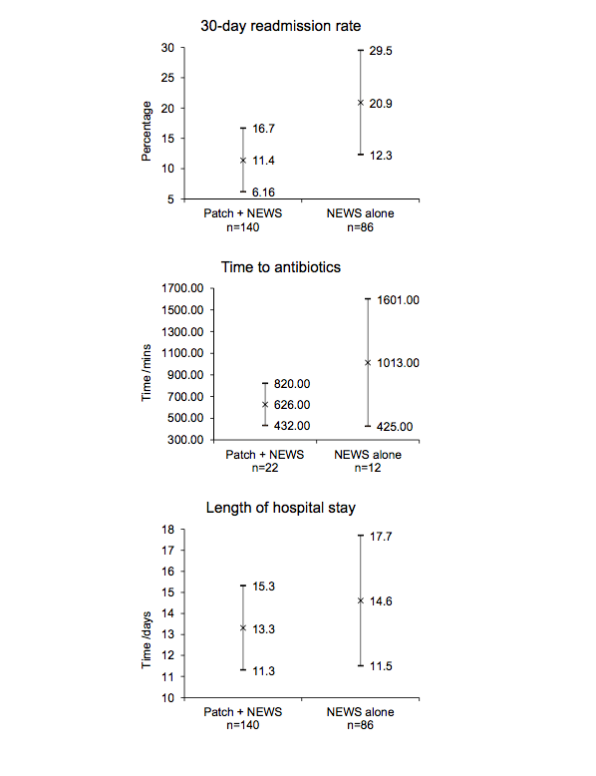
Scatter graphs to show mean (x) and 95% CIs between trial arms for time to antibiotics in sepsis, length of hospital stay, and 30-day readmission rate. NEWS: National Early Warning Score.

Table 2 summarizes the main results of the study. In the intervention arm, 17.1% (24/140) of patients experienced a sepsis event; this figure was 14% (12/86 patients) in the control arm. Of 36 sepsis events recorded in randomized bays, there were sufficient data to analyze the time to antibiotics in 34 cases. The average time from the first evidence of sepsis to the first administration of antibiotics was 626 minutes in the intervention group (n=22, 95% CI 431.7-820.3 minutes). The average time to antibiotics in the control group was 1012.8 minutes (n=12, 95% CI 425.0-1600.6 minutes; Figure 3). Of 36 sepsis events, 34 cases were triggered by derangements in the heart rate, respiratory rate, or temperature—heart rate alone (n=1); temperature alone (n=1); heart rate and temperature (n=23); respiratory rate and temperature (n=2); heart rate, respiratory rate, and temperature (n=7); and unknown (n=2).

### Secondary Outcome Measures

There were very few inpatient deaths (n=1) and admissions to level II/III care (n=5) across both study arms. The length of hospital stay was on average 1.3 days shorter in patients who had continuous monitoring (13.3 days, 95% CI 11.3-15.3 days vs 14.6 days, 95% CI 11.5-17.7 days). The rate of readmissions within 30 days of discharge was lower in the continuous monitoring group (11.4%, 95% CI 6.16-16.7 vs 20.9, 95% CI 12.3-29.5; Figure 3).

### Exploratory Analysis

When the 2 nonrandomized bays were analyzed alongside the 6 randomized bays, the results were very comparable with narrower CIs (see Multimedia Appendix 3).

### Patient Acceptability and Compliance

**Figure 4:**
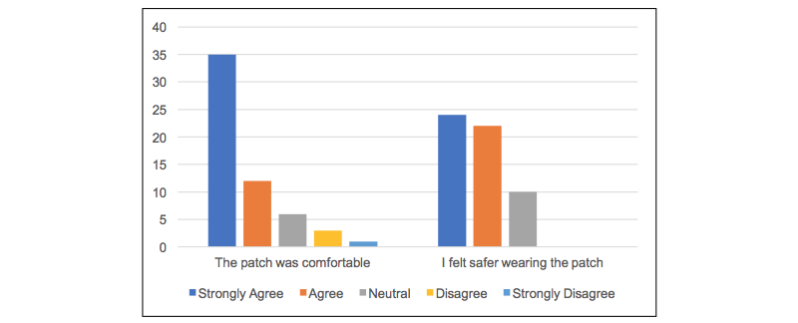
Patient responses to the questionnaire.

Overall, 41% (58/140) patients in the continuous monitoring group returned a short questionnaire; the results are shown in Figure 4. The majority of patients found the patch to be comfortable (47/57, 82%) and reported feeling safer while wearing the patch (46/56, 82%).

Patients in the continuous monitoring group wore the patch for an average of 5 (range: 1-24) days. Of 142 patients who wore the monitoring patch, 34 had the continuous monitoring discontinued early (Figure 2); 23 of these were at patient request. Two patients developed a rash under the electrodes, 18 patients found it itchy or bothersome, and 3 patients did not offer a reason for removing the patch.

### Progression Criteria

In the pilot trial, 350 patients were recruited within 7 months, which is well within the recruitment target. Adherence to protocol was acceptable; 75.7% (106/140) of patients in the intervention arm wore the patch for at least 5 days.

The low rate of sepsis events across both arms of the study has meant that the CIs around the mean time to antibiotics are wide, and it has not been possible to accurately estimate the intercluster correlation coefficient for this endpoint from the study data. As such, it is unlikely that the time to antibiotics in cases of sepsis is a suitable outcome measure to inform the sample size of a definitive trial using the same protocol.

**Table 1:** Baseline patients’ characteristics.

Characteristics		SensiumVitals + intermittent monitoring (n=140)	Intermittent monitoring alone (n=86)
Males, n (%)		76 (54.3)	39 (45.4)
Females, n (%)		64 (45.7)	47 (54.6)
Age (years), mean, range		65.2, 24-94	63.7, 21-92
**American Society of Anaesthesiologists score for preoperative functional status, n (%)**			
	1	9 (6.4)	9 (10.5)
	2	62 (44.3)	35 (40.7)
	3	42 (30.0)	22 (25.6)
	4	3 (2.1)	3 (3.5)
	Not documented	24 (17.1)>	17(19.8)
Emergency admissions, n (%)		70 (50)	44 (51.2)
Elective admissions, n (%)		70 (50)	42 (48.8)
Surgical intervention, n (%)		103 (73.6)	62 (72.1)
Medical outliers, n (%)		19 (13.6)	14 (16.3)

**Table 2:** A summary of outcome measures.

Outcome measures		SensiumVitals + intermittent monitoring (n=140)	Intermittent monitoring alone (n=86)
Complications^a^, n (%)		102 (72.9)	57 (66.3)
Major complications^b^, n (%)		8 (5.7)	5 (5.8)
Sepsis events, n (%)		24 (17.1)	12 (14.0)
Time (min) to antibiotics in cases of sepsis^c^, mean (95% CI)		626.0 (431.7-820.3)	1012.8 (425.0-1600.6)
**Level II or III admissions**			
	n (%)	3 (2.1)	2 (2.3)
	95% CI	0-4.54	0-5.51
Length of stay (in days) in hospital, mean (95% CI)		13.3 (11.3-15.3)	14.6 (11.5-17.7)
**Readmissions**			
	n (%)	16 (11.4)	18 (20.9)
	95% CI	6.16-16.7	12.3-29.5
Inpatient deaths, n (%)		1 (0.7)	0 (0)

^a^All.

^b^Clavien-Dindo>2.

^c^SensiumVitals + intermittent monitoring (n=22); intermittent monitoring alone (n=12)

## Discussion

In this single-center, randomized controlled pilot trial, surgical patients with evidence of sepsis tended to receive antibiotics faster if they received continuous vital signs monitoring compared with those receiving usual intermittent monitoring alone. Patients receiving continuous vital signs monitoring had a shorter average length of hospital stay and were less likely to require readmission within 30 days of discharge. Patients found the monitoring device to be acceptable in terms of comfort and perceived safety.

The findings must be interpreted within the limitations of the study. A formal sample size calculation was not possible given the lack of data surrounding the primary outcome measure, and so the findings were limited to descriptive statistics; no formal statistical comparison was possible [14]. Although the wide, overlapping CIs suggest that a statistically significant difference between the 2 groups is unlikely, with a larger sample size and increased study power, it is possible that the observed trends might become statistically significant. In addition, the relatively small number of sepsis cases means there is likely to be an imbalance in prerandomization variables, which would require an adjustment in a formal analysis.

There were very few cases of inpatient mortality or admission to level II/III care, making comparisons between the monitoring arms difficult. One explanation for this low event rate is that the population contained a high proportion of low-risk patients—medical outliers and those who did not undergo surgery during their admission. A more striking effect might be evident in a higher-risk population.

The limitations of the randomization technique must also be considered. Ideally, the study data would have been analyzed at the cluster level, but small numbers of patients within each bay necessitated analysis at the individual level. The cluster-randomization methodology led to differences in the baseline demographics of the treatment arms. One of the female bays allocated to receive continuous monitoring had a proportionally lower turnover of patients than the other bays. This led to an imbalance in the male:female ratio between the 2 arms. The fact that the control arm was, on average, 1.5 years younger than the treatment arm may have conferred an advantage to this group.

The potential benefits of continuous monitoring may have been underestimated in this study owing to the exposure to the patch in the intervention arm. Nearly a quarter of the patients who were allocated to receive continuous monitoring did not wear the patch for their entire admission; however, this may reflect what can be truly expected in the clinical environment. The patient-reported acceptability of the device was high in the questionnaire results. This result may be subject to selection bias. A number of patients were missed when they were discharged from hospital outside normal working hours, and enthusiastic patients may have been more likely to complete the questionnaire.

There were other challenges to implementing the technology. There was initially an unacceptably high number of alerts sent to nursing staff; these were reduced by 90% by adjusting the alarm thresholds to more clinically appropriate levels and increasing the intervals between reminder alerts. Engagement with the new system varied between nursing staff but was aided by support from senior ward nurses. Engagement was further increased with the implementation of changes suggested by the nursing staff themselves such as smaller devices and louder alert tones.

There are few clinical evaluations of continuous vital signs monitoring in the literature [8]. The preponderance of observational studies means that causal associations between interventions and patient outcomes have to be interpreted with care. The 3 largest randomized controlled trials of continuous monitoring report conflicting results, illustrating the difficulties in evaluating such complex interventions. The potential benefit of the additional monitoring may be negated by inadequacies in other areas, such as staffing levels, escalation protocols, and nursing compliance [15]. Demonstrating clinical benefit will likely require large, well-controlled studies in high-risk populations to find significant differences in clinical outcomes such as critical care admissions. This is important as these systems are not without financial cost. System prices are around US $1500, and the cost of disposable patches varies [7]. Further research is required to determine with certainty whether continuous postoperative monitoring offers a significant benefit over intermittent monitoring and can be justified for routine care in terms of cost-effectiveness.

In conclusion, this study has demonstrated the practicability and acceptability of implementing a remote continuous monitoring system in the general surgical ward setting. There is a trend toward clinical benefit. The findings of this study will be used to inform the protocols for further evaluations. Follow-up studies should be individually randomized and stratified to minimize the baseline differences between the 2 treatment arms and include a high-risk population with a high rate of adverse events. Furthermore, rare outcomes, such as mortality, should be avoided in preference of endpoints that are common to all participants such as the length of hospital stay.

## Appendix

Multimedia Appendix 1 CONSORT-EHEALTH checklist (V 1.6.1).

Multimedia Appendix 2 Patient information sheet.

Multimedia Appendix 3 Exploratory analyses and questionnaire.

## Abbreviations

CONSORT: Consolidated Standards of Reporting Trials
NIHR: National Institute for Health Research
