# Supplementary material for: Continuous Versus Intermittent Vital Signs Monitoring Using a Wearable, Wireless Patch in Patients Admitted to Surgical Wards: Pilot Cluster Randomized Controlled Trial
Source: J Med Internet Res. 2018 Dec 11;20(12):e10802. doi: 10.2196/10802 (PMC6305881; doi:10.2196/10802)
Supplement: Multimedia Appendix 2 [file jmir_v20i12e10802_app2.pdf]

The Leeds Teaching Hospitals NHS Trust

IRAS reference number: 204340

John Goligher Colorectal Unit

Research Office

Ground Floor Lincoln Wing
St James University Hospital
Beckett Street
Leeds
LS9 7TF
Tel: 0113 20 64672

Version 3

An evaluation of remote, near-continuous vital signs monitoring in patients admitted to general surgical wards

PARTICIPANT INFORMATION SHEET AND INFORMED CONSENT DOCUMENT

A large-print version of this sheet is available on request.

You have been invited to take part in a research study. Before you decide if you want to take part, we would like to explain why the research is being done, how we will use the information we have about you, and what the study will involve.

Please read this information carefully, and discuss it with others if you like. Ask us if anything is unclear, or if you would like more information.

Once you have read this information, the study team will talk to you about the study again and you can ask any questions you like.

Part 1 tells you the purpose of the study and what will happen if you take part.

Part 2 gives you more detailed information about the conduct of the study.

Take time to decide whether or not you wish to take part.

Thank you for reading this information sheet.

Part 1

What is the purpose of the study?

This study will investigate the use of remote, near-continuous monitoring in patients admitted to one of four general surgical wards at St. James's University Hospital, Leeds.

Unfortunately, despite the best efforts of the clinical team, up to a third of patients who undergo major surgery will experience a significant complication. These often include infections, such as pneumonia or wound infections, which require urgent antibiotics.

One of the ways patients are monitored for complications is by charting their vital signs: blood pressure, heart rate, breathing rate and temperature. The nurse looking after the patient will usually check these signs every few hours in the days after surgery. The vital signs are used to form a score, the National Early Warning Score (NEWS), which can alert if the patient becomes unwell.

The SensiumVitals® monitoring system measures heart rate, breathing rate and temperature continuously, and sends the information to a mobile phone carried by the nurse every two minutes. It is a wearable, wireless patch that is applied to the chest after surgery, and alerts the nurse if the patient's vital signs become abnormal. This could help detect unwell patients earlier than traditional NEWS monitoring.

In order to test this theory, a study will be done comparing the SensiumVitals® system with NEWS monitoring. The main aim is to provide important information about whether the patch works and if it improves outcomes for patients admitted to surgical wards.

Why have I been invited?

Four general surgery wards at St. James's University Hospital are taking part in the study. You will be asked to join the study if you are admitted to one of these wards, regardless of whether you are having surgery or not. You will be given information about the study and you will be allowed time to decide whether you would like to take part.

Do I have to take part?

No. It is up to you to decide whether or not to take part. If you decide to take part you will be given this information sheet to keep and be asked to sign a consent form to confirm that you understand what is involved when taking part in this study. If you decide to take part you are free to leave the study at any time and without giving a reason. If you withdraw, unless you object, we will still keep records relating to the treatment given to you, as this is valuable to the study. A decision to withdraw at any time, or a decision not to take part, will not affect the quality of care you receive. In the unlikely event that, during the study, you are no longer capable of agreeing to take part, you will remain in the study until you are well enough to give permission again. Your close relatives' views of your wishes may be taken into account.

What will happen to me if I take part?

If you choose to take part in the study your care in hospital will not differ from standard care.

Patients who enter the study will be randomly allocated to one of two study groups. All of the patients who enter the study will receive standard NEWS monitoring, as usual. Half of the patients who enter the study will also receive the SensiumVitals® monitoring. This decision is made at random, and it is not possible for you or the clinical team to change it.

If you are in the SensiumVitals® group, you will be asked to wear the patch on your chest for the whole of your admission, in addition to receiving usual NEWS monitoring. You will be given a full explanation of the SensiumVitals® remote monitoring system at this time.

Everyone who enters the study will be followed during the course of their hospital stay, including any operation they have.

Information about your hospital stay will be collected by the research team. This information will include any complications you experience, including infections, and how quickly they are treated. You will also be followed up if you are moved to a high-dependency ward. Information will be gathered about how long you stay in hospital.

An important part of the study will be to assess how patients and nurses feel about the SensiumVitals® system. If you receive the SensiumVitals® monitoring system, you will be invited to fill out a questionnaire and undertake a short interview (15-30 minutes) at the end of your hospital admission.

Once you are discharged form hospital, your participation in the trial will be over. If you like, the results of the study can be sent to you at a later date, either by email or by post, according to your preference.

What do I have to do?

After you have had time to consider if you would like to participate in the trial, a member of the team will approach you to find out your decision.

What is being tested?

We are testing a device that continuously monitors vital signs: heart rate, breathing rate and temperature. This information is transferred remotely to a mobile phone carried by the nurse, which alerts if the vital signs stray beyond normal parameters.

It is thought that continuous monitoring might help detect complications early, but not enough is known about this technology to say for sure. This is why it has to be tested against the current national standard of care: NEWS monitoring.

All data will be anonymised and coded. It will only be accessible to the researchers.

What are the alternatives for diagnosis or treatment?

Whether or not you choose to take part in the study you will receive the standard treatment: NEWS monitoring. This study will not change any further treatment you may require during or after your hospital stay.

What are the possible disadvantages and risks of taking part?

If you are in the group that receives standard NEWS monitoring alone, your care will not vary from that of someone who is not taking part in the research, although information about your hospital stay will be collected.

If you are in the group of patients that receives the SensiumVitals® patch, you will be required to have the patch applied when you are admitted to the ward. This process is painless, but will take about 10 minutes and may involve some skin preparation of the area on the chest where the patch is applied. This sometimes includes shaving small areas for the patch to stick to. The patch's battery lasts for five days. You will be expected to wear a patch for the whole of your hospital stay. This may mean getting the patch changed a number of times, if you are in hospital for a few weeks.

Once you are wearing the patch, you are free to move about as normal. The patch is not connected to any machines and so it should not limit your movement.

It is important to remember that your doctors and nurses may not be able to detect complications even if you are wearing the patch, so if you are feeling unwell or have any concerns you should alert a member of staff.

At the end of your hospital stay, the patch will be removed and you will be asked to fill out a questionnaire. You will also be invited to undergo a short interview before going home and/or return to the hospital at a later date to take part in two focus groups, lasting an hour each. If you are not comfortable sharing your views about the patch, you may decline these. The interviews and the focus groups will be audio recorded. If you decide to take part in the focus groups, you will be reimbursed for your time and travel expenses.

What are the possible benefits of taking part?

If you receive the SensiumVitals® monitoring, there is the possibility that any complications you experience may be detected earlier. However, although you may enjoy wearing the monitoring device, participating in this study may not directly benefit your health during your stay in hospital. Information gathered from this study may benefit future patients and pave the way for improved care.

What happens when the research study stops?

Your involvement in this study will stop once you are discharged from hospital. After this time your follow up will be as standard treatment. This will vary according to your needs and the reason for your admission.

What if there is a problem?

If you have a concern about any aspect of this study, you should ask to speak with the researchers who will do their best to answer your question. If you remain unhappy and wish to complain formally, you can do this through the NHS Complaints Procedure. Details can be obtained from the hospital.

In the event that something does go wrong and you are harmed during the research study there are no special compensation arrangements. If you are harmed and this is due to someone's negligence then you may have grounds for a legal action for compensation but you may have to pay your legal costs. The normal National Health Service complaints mechanisms will still be available to you.

If you have a complaint about the care you have received you can contact the Patient Advice and Liaison Service (PALS) at:
Patient Relations Department
Trust Head Quarters
St James's Hospital
Leeds LS9 7TF
Tel 0113 2066261
Email: patient experience.leedsth@nhs.net

Will my taking part be kept confidential?

If you decide to participate in this study the information collected about you will be handled strictly in accordance with the consent that you have given and also the 1998 Data Protection Act. Please refer to Part 2 for further details.

Contact Details

If you have any further questions about your illness or clinical studies, please discuss them with your doctor.

If you would like further information about clinical research, the UK Clinical Research Collaboration (a partnership of organisations working together on clinical research in the UK) have published a booklet entitled 'Understanding Clinical Trials'. Contact UKCRC: Tel: +44 (0)207 670 5452; website www.ukcrc.org

Your contact telephone numbers:

…………………………………………………………………………………………

…………………………………………………………………………………………

This completes Part 1 of the Information Sheet. If the Information in Part 1 has interested you and you are considering participation, please continue to read the additional information in Part 2 before making any decision.

Part 2

What if new information becomes available?

Sometimes during clinical research, new information becomes available regarding the systems being studied. If this happens, we will tell you about it and discuss with you whether you want to or should continue in the study. If you decide to withdraw, we will make arrangements for your care to continue. If you decide to continue in the study you will be asked to sign an updated consent form.

On receiving new information, we might consider it to be in your best interests to withdraw you from the study. If so, we will explain the reasons and arrange for your care to continue.

If the study is stopped for any other reason, you will be told why and your continuing care will be arranged.

What will happen if I don't want to carry on with the study?

If you withdraw consent from further study treatment, and/or follow-up, your data will remain on file and will be included in the final study analysis.

If you leave the study and do not wish for any further information to be collected, you should inform your clinical care team of this in order that no further follow-up information is collected from your medical records.

Please note the study team may be required to continue to collect some limited information about you in the case of any unwanted effects you may have as a result of taking part in the trial. This will only be collected if required by the regulatory authorities. In line with Good Clinical Practice guidelines, at the end of the study, your data will be securely archived for a minimum of 5 years. Arrangements for confidential destruction will then be made.

Will my taking part in this study be kept confidential?

If you decide to participate in this study, the information collected about you will be handled in accordance with the consent that you have given and also the 1998 Data Protection Act.

- The information needed for study purposes will be recorded on paper forms and collected by the researchers at St James's University Hospital, Leeds. These forms will be kept for five years and then destroyed. Access to the forms will be limited to the researchers involved in the study.
- You will be allocated a study number, which will be used along with your initials to identify you on each paper form. Your full name will be included on your consent form and a copy of this will be collected by the researchers and stored securely at St James's University Hospital.
- Every effort will be made to ensure that any further information about you that leaves the hospital will have your name and address removed so that you cannot

be recognised from it; this information will usually be removed by a member of the study team at your hospital.

- Any audio recordings will be anonymised and destroyed once the relevant information has been obtained.
- If you would like to be informed of the results of the study, your contact details will be collected and stored securely.

Your data will be entered onto a secure database held on an encrypted laptop belonging to the University of Leeds in accordance with the 1998 Data Protection Act.

Any of your data uploaded to the SensiumVitals® system will be linked to your name for clinical purposes. However, Sensium Healthcare will not have access to any personal clinical information about you. Data uploaded to the SensiumVitals® system will be downloaded onto a secure computer held at St James's University Hospital at the end of the study and deleted from the SensiumVitals® system.

Your healthcare records may be looked at by authorised individuals from the research team, Leeds Teaching Hospitals NHS Trust, the University of Leeds or the regulatory authorities to check that the study is being carried out correctly.

Informing your General Practitioner (GP)

Your GP, and the other doctors involved in your healthcare, will be informed of your participation in this study.

What will happen to the results of the research study?

When the study is complete the results may be published in a medical journal, but no individual participants will be identified. If you would like to obtain a copy of the published results, please ask your doctor.

Contact for further information

You are encouraged to ask any questions you wish, before, during or after your treatment. If you have any questions about the study, please speak to your study nurse or doctor, who will be able to provide you with up to date information about the procedure(s) involved. If you wish to read the research on which this study is based, please ask your study nurse or doctor. If you require any further information or have any concerns while taking part in the study please contact one of the following people:

…………………………………………………………………………………………

…………………………………………………………………………………………

If you decide you would like to take part then please read and sign the consent form. You will be given a copy of this information sheet and the consent form to keep. A

copy of the consent form will be filed in your patient notes, one will be filed with the study records and one may be sent to the Research Sponsor.

You can have more time to think this over if you are at all unsure.

Thank you for taking the time to read this information sheet and to consider this study.
