# Supplementary material for: Continuous Versus Intermittent Vital Signs Monitoring Using a Wearable, Wireless Patch in Patients Admitted to Surgical Wards: Pilot Cluster Randomized Controlled Trial
Source: J Med Internet Res. 2018 Dec 11;20(12):e10802. doi: 10.2196/10802 (PMC6305881; doi:10.2196/10802)
Supplement: Multimedia Appendix 3 [file jmir_v20i12e10802_app3.pdf]

Supplementary material

		SensiumVitals® +NEWS *(n=140)*	NEWS alone *(n=86)*
Number of patients with complications (all[a])		100 (71.4%)	57 (66.3%)
	Anastomotic leak	1	1
	Bowels not opened	7	5
	Chest pain	3	2
	Confusion	3	1
	Electrolyte disturbance	8	2
	Increased stoma output	1	2
	Loose stools	3	0
	Nausea and vomiting	10	8
	Pain	4	5
	Pyrexia	5	1
	Sepsis	24	12
	Rectal bleeding	1	2
	Respiratory complication	3	4
	Stroke	1	0
	Urinary tract infection	5	2
	Wound complication	3	3
	Other	18	7
Number of patients with major complications (Clavien-Dindo >2[b])		8 (5.7%)	5 (5.8%)
Sepsis events		24 (17.1%)	12 (14.0%)
	Intra-abdominal collection	4	0
	Ischaemic bowel	1	0
	Venous access infection	1	1
	Respiratory tract infection	3	3
	Urinary tract infection	2	1
	Wound infection	5	2
	Unknown source	7	4
	Other	1	1

[a]All complications includes any deviations from the normal post-operative course, from minor (such as nausea) to major (such as death).
[b]A Clavien-Dindo score >2 indicates that that the complication required critical care admission or further surgical/radiological intervention, or resulted in death.

Complication rates between intervention and control arms

	**SensiumVitals +NEWS** *(n=140)*	**NEWS alone: incl non-randomised bays** *(n=210)*	**NEWS alone: only randomised bays** *(n=86)*
Males	76 (54.3%)	77 (36.7%)	39 (45.4%)
Females	64 (45.7%)	133 (63.3%)	47 (54.6%)
Age			
Mean	65.2 years	60.5 years	63.7 years
Range	24-94 years	19-93 years	21-92 years
ASA			
1	9 (6.4%)	19 (9.0%)	9 (10.5%)
2	62 (44.3%)	97 (46.2%)	35 (40.7%)
3	42 (30.0%)	50 (23.8%)	22 (25.6%)
4	3 (2.1%)	5 (2.4%)	3 (3.5%)
Not documented	24 (17.1%)	39 (18.6%)	17(19.8%)
Emergency admissions	70 (50%)	113 (53.8%)	44 (51.2%)
Elective admissions	70 (50%)	97 (46.2%)	42 (48.8%)
Surgical intervention	103 (73.6%)	154 (73.3%)	62 (72.1%)
Medical outliers	19 (13.6%)	27 (12.9%)	14 (16.3%)
Number of complications (all[a])	102 (72.9%)	143 (68.1%)	57 (66.3%)
Number of major complications (Clavien-Dindo >2[b])	8 (5.7%)	17 (8.1%)	5 (5.8%)
Sepsis events	24 (17.1%)	33 (15.7%)	12 (14.0%)
Time to antibiotics in cases of sepsis	n=22	n=32	n=12
Mean	626.0 minutes	900.0 minutes	1012.8 minutes
95% confidence interval	431.7-820.3	621.6-1178.4	95% CI 425.0-1600.6
Level II/III admissions	3 (2.1%)	5 (2.4%)	2 (2.3%)
95% confidence interval	0%-4.54%	0.319%-4.44%	0%-5.51%
Length of stay in hospital			
Mean	13.3 days	15.5 days	14.6 days

	95% confidence interval	11.3-15.3	10.1-20.9	11.5-17.7
Readmissions		16 (11.4%)	38 (18.1%)	18 (20.9%)
	95% confidence interval	6.16%-16.7%	12.9%-23.3%	12.3%-29.5%
Inpatient deaths		1 (0.7%)	0 (0%)	0 (0%)

[a] All complications include any deviations from the normal post-operative course, from minor (such as nausea) to major (such as death).
[b] Clavien-Dindo is a scale for the severity of complications. A Clavien-Dindo score >2 indicates that that the complication required critical care admission or a further surgical procedure, or resulted in death.

Summary of outcome measures including exploratory analysis

Statement	Strongly Agree	Agree	Neutral	Disagree	Strongly Disagree
**Comfort**					
The SensiumVitals® Patch was comfortable to wear.					
**Quality of Care**					
I felt safer because my vital signs were being monitored constantly.					
**We welcome any additional comments you may have:**					

Patient questionnaire
